# RecPhyloXML: a format for reconciled gene trees

**DOI:** 10.1093/bioinformatics/bty389

**Published:** 2018-05-14

**Authors:** Wandrille Duchemin, Guillaume Gence, Anne-Muriel Arigon Chifolleau, Lars Arvestad, Mukul S Bansal, Vincent Berry, Bastien Boussau, François Chevenet, Nicolas Comte, Adrián A Davín, Christophe Dessimoz, David Dylus, Damir Hasic, Diego Mallo, Rémi Planel, David Posada, Celine Scornavacca, Gergely Szöllősi, Louxin Zhang, Éric Tannier, Vincent Daubin

**Affiliations:** 1 Univ Lyon, Université Lyon 1, CNRS, Laboratoire de Biométrie et Biologie Évolutive UMR5558, F-69622 Villeurbanne, France; 2 INRIA Grenoble Rhône-Alpes, F-38334 Montbonnot, France; 3 MTA-ELTE Lendület Evolutionary Genomics Research Group, Budapest, Hungary; 4 Department of Biological Physics, Eötvös Loránd University, Budapest, Hungary; 5 LIRMM, Université de Montpellier, CNRS, Montpellier, France; 6 Institut de Biologie Computationnelle (IBC), Montpellier, France; 7 Department of Mathematics, Stockholm University, Stockholm, Sweden; 8 Swedish e-Science Research Centre (SeRC), Stockholm, Sweden; 9 Department of Computer Science and Engineering, University of Connecticut, Storrs, CT, USA; 10 Institute for Systems Genomics, University of Connecticut, Storrs, CT, USA; 11 ISEM, CNRS, Université de Montpellier, IRD, EPHE, Montpellier, France; 12 MIVEGEC, CNRS 5290, IRD 224, Université de Montpellier, Montpellier, France; 13 Department of Computational Biology, University of Lausanne, Lausanne, Switzerland; 14 Center for Integrative Genomics, University of Lausanne, Lausanne, Switzerland; 15 Department of Genetics, Evolution and Environment, University College London, London, UK; 16 Department of Computer Science, University College London, London, UK; 17 Swiss Institute of Bioinformatics, Lausanne, Switzerland; 18 Department of Mathematics, Faculty of Science, University of Sarajevo, Sarajevo, Bosnia and Herzegovina; 19 Virginia G. Piper Center for Personalized Diagnostics, Biodesign Institute, Arizona State University, Tempe, AZ, USA; 20 Laboratoire d’Analyse Bio-informatique en Génomique et Métabolisme CNRS-UMR 8030, Commissariat à l’Énergie Atomique (CEA), Institut de Génomique, Genoscope, Evry, France; 21 Department of Biochemistry, Genetics and Immunology, University of Vigo, Vigo, Spain; 22 Department of Mathematics, National University of Singapore, Singapore, Singapore

## Abstract

**Motivation:**

A reconciliation is an annotation of the nodes of a gene tree with evolutionary events—for example, speciation, gene duplication, transfer, loss, etc.—along with a mapping onto a species tree. Many algorithms and software produce or use reconciliations but often using different reconciliation formats, regarding the type of events considered or whether the species tree is dated or not. This complicates the comparison and communication between different programs.

**Results:**

Here, we gather a consortium of software developers in gene tree species tree reconciliation to propose and endorse a format that aims to promote an integrative—albeit flexible—specification of phylogenetic reconciliations. This format, named recPhyloXML, is accompanied by several tools such as a reconciled tree visualizer and conversion utilities.

**Availability and implementation:**

http://phylariane.univ-lyon1.fr/recphyloxml/.

## 1 Introduction

The relationships between the history of genomes or species and the history of their constituent genes are often described through reconciliation. A reconciliation consists of an association between the nodes of a gene tree and the nodes or branches of a species tree, along with different evolutionary events undergone by the gene. For comprehensive reviews on the subject of reconciliations and their inference, see for example Nakhleh (2013) or Szöllősi *et al.* (2015).

Reconciliations can be used to understand the history of a specific gene family, and to study the evolutionary and functional relationships between several families. They can also be used to infer genome-wide parameters such as rates of gene duplication, loss, or lateral gene transfers (Sjöstrand *et al.*, 2014; Szöllősi *et al.*, 2013a), or population parameters such as divergence time and ancestral population size (Dutheil *et al.*, 2009). Furthermore, reconciliation based metrics can be used as a criterion to construct better gene trees (Durand *et al.*, 2006; Scornavacca *et al.*, 2013; Sjöstrand *et al.*, 2014; Szöllősi *et al.*, 2013a; Wu *et al.*, 2013) or better species tree (Boussau *et al.*, 2013; Nakhleh, 2013).

There are many algorithms and software to infer reconciliations (Nakhleh, 2013; Szöllősi *et al.*, 2015), and while they share many features, each has some unique characteristics.

Some methods work according to a parsimony principle [see for instance Durand *et al.* (2006), Bansal *et al.* (2012) and Chan *et al.* (2017)] while others rely on a probabilistic approach (Åkerborg *et al.*, 2009; Sjöstrand *et al.*, 2014; Szöllősi *et al.*, 2013a). Reconciliation methods may differ in the type of events they consider. Some methods also require a dated species tree (i.e. a species tree where the relative timing of internal speciations is known) while others do not.

The fact that reconciliation programs (or rather each program family) use different formats to represent reconciliations makes it difficult to compare, switch between or use together reconciliations inferred from different pieces of software, which can hamper proper comparison and validation studies. This also means that any post-analysis or visualization software will either have limited scope (it will only be able to take as input the reconciliations of specific pieces of software) or be burdened by the implementation of readers for several formats.

In this paper, we aim to propose a generic reconciliation format encompassing the specificities of different reconciliation programs. This will make reconciliation based analysis more accessible to scientists without the need to develop or use multiple format conversion scripts.

In order to include all properties described in the scientific literature about gene tree species tree reconciliation, we should first be able to annotate gene tree nodes with events related to species tree nodes, such as speciations, and events associated to species tree branches, such as gene duplication (D), gene loss (L), lateral gene transfer (T), transfer with replacement (TR), gene conversion (C) and incomplete lineage sorting (ILS) (Mallo *et al.*, 2014; Rasmussen and Kellis, 2012; Than *et al.*, 2008). Reconciliations can be carried out with dated or undated species trees. In a dated species tree, the relative order of speciations is known and it would be desirable to be able to include information about the relative time at which the different events occurred in the reconciliation.

Transfers are written with two separate events: a gene lineage leaving a species tree branch (branching out) and then entering another species tree branch (transfer reception). As noted in Szöllősi *et al.* (2013b), most transfers originate from extinct or unsampled lineages (i.e. branches absent from the species tree). This implies that the bifurcation in the gene tree when a lineage leaves the species tree is not the transfer itself but actually a speciation toward an unsampled/extinct lineage. Our format nevertheless reflects the generality of this event by adopting a neutral label compatible with the different representations of transfers.

Moreover, this notion of evolution in unsampled lineages implies the possibility of a bifurcation in the gene tree in such a lineage. The children of the bifurcation can undergo transfers back to the sampled lineages. The unseen bifurcation might be a duplication, a speciation or a transfer between two unsampled lineage. Existing models are yet unable to discriminate these events. This idea is reflected in our format thanks to a specific way to specify a bifurcation in an unsampled lineage.

There have been previous attempts to develop formats able to represent evolutionary events along a phylogeny. The PhyloXML format (Han and Zmasek, 2009) is able to depict various annotations along a tree. It already has some way of representing evolutionary events along a phylogeny, but with limitations. For example PhyloXML lacks a mean to specify the species associated with the different events and only includes a rudimentary representation of transfers.

Adapting the already existing tags for evolutionary event in PhyloXML would require a near complete overhaul; rather, we propose a new format (recPhyloXML) with entirely new tags, ensuring no confusion with PhyloXML.

### 1.1 State of the art

Existing reconciliation formats can be broadly categorized in two groups.

The first group describes reconciliation events as labels in a Newick or NHX tree, in place of the nodal support (e.g. bootstrap) information or in a devoted NHX comment field. Programs like ALE (Szöllősi *et al.*, 2013a), NOTUNG (Durand *et al.*, 2006; Stolzer *et al.*, 2012), DrML (Górecki and Eulenstein, 2014), phylotoo2 (Zheng and Zhang, 2017) or PrIME (Åkerborg *et al.*, 2009; Sjöstrand *et al.*, 2014) adhere to this group. The Newick-based reconciliation formats have the advantage of representing the phylogeny. However the reconciliation information often takes the space of other measures like bootstrap values [as in Szöllősi *et al.* (2013a), or Górecki and Eulenstein (2014)]. The NHX-based format solves this by allocating a specific space for the reconciliation. A common problem with NHX and Newick-based formats is that some characters are forbidden in the leaf names and annotations (These forbidden characters are : , : () ; [] in Newick and NHX.), while sometimes species or gene annotations contain these characters (whereas they rarely contain whole XML tags). In addition, there is no formal format for information contained in NHX comment fields; thus, this information may not be accessible across software platforms.

The second group represents reconciliations as lists of gene tree nodes mapping to species tree nodes, making references to an implicit or external gene tree (meaning that the gene tree structure might not be included in the reconciliation). Examples of such output formats are used by ranger-DTL (Bansal *et al.*, 2012), ecceTERA (Jacox *et al.*, 2016), DLcoalRecon (Rasmussen and Kellis, 2012), Mowgli (Doyon *et al.*, 2010), the visualization software SylvX (Chevenet *et al.*, 2016) or the simulation software SimPhy (Mallo *et al.*, 2016).

## 2 Format presentation

To describe reconciliations, we present recPhyloXML, recGeneTreeXML, recSpeciesTreeXML, three grammars extending the PhyloXML format. We also introduce recGeoXML, a grammar to annotate reconciliations with geographic information.

They both rely on an XML structure composed of hierarchical tags. A specific tag may have different attributes which can be obligatory or optional.

In this section we briefly detail the structure of the PhyloXML used in our format. We then expand on the tags that are specific to reconciliations.

### 2.1 Elements in common with PhyloXML

In PhyloXML, a tree is delimited by the tag <phylogeny> </phylogeny>, which is included in a <phyloxml> </phyloxml> root tag that specifies that the file follows the PhyloXML format. Inside the <phylogeny> </phylogeny> tag, each clade is recursively inscribed in a <clade> </clade> tag. This clade tag possesses a facultative attribute to describe branch length. The name or identifier of the node is given in the <name> </name> tag. Further information can be included such as support value (<confidence></confidence>) or miscellaneous information (<description></description>).

### 2.2 New elements

In our format, a reconciliation (<recPhylo> tag) is defined as a set comprised of one or more reconciled gene trees (<recGeneTree> tag), and a species tree (<spTree> tag). These tags are described in the next section. Also, reconciled gene trees are always rooted and this is specified by using the tag

<phylogeny rooted=“true”></phylogeny>.

A recPhyloXML file allows you to store and share one or more reconciled genes trees and the associated species tree. A recGeneTreeXML file allows you to add a list of evolutionary events to the description of gene tree nodes (otherwise referred to as clades in PhyloXML), possibly also containing detailed geographic information thanks to the recGeoXML grammar (<geography> tag). This tag can also be used in a recSpeciesTreeXML file that currently differs from PhyloXML file only in this point.

### 2.3 recGeneTreeXML

recGeneTreeXML enriches the PhyloXML vocabulary by adding the complex tag <eventsRec> that must be included inside a <clade> tag.

The <eventsRec> tag contains the sequence of evolutionary events that occur along a gene tree branch.

Each type of evolutionary event is represented by a specific tag. These can be of two types, according to whether they concern a branch or a node of the gene tree:

- **Non terminal event:**<transferBack>. This tag can be used as many times as necessary. This event does not cause any bifurcation in the gene tree.
- **Terminal events:**<speciation>, <branchingOut>, <bifurcationOut>, <duplication>, <loss> and <leaf>. There is exactly one of these tags at the end of the sequence of events contained in the <eventsRec> tag.

Terminal events cause either a bifurcation in the gene tree (<speciation>, <branchingOut>, <bifurcationOut>, <duplication>) or the end of a lineage (<leaf>, <loss>).

Aside from the <bifurcationOut> and <transferBack> tags, all tags have an obligatory speciesLocation attribute that specifies in which species the event takes place. For <bifurcationOut>, the event always takes place in an unsampled/extinct lineage. <transferBack> events have instead a destinationSpecies attribute that specifies the species that receives the transfer. All event tags also have a facultative confidence attribute that is intended to store a support value for this event (Nguyen *et al.*, 2013). Additionally, all event tags have a facultative timeSlice attribute that can, in models where the species tree is dated and subdivided for instance [as done for example in Doyon *et al.* (2010)], provide information on the timing of the event. Finally, the <leaf> tag has a facultative geneName attribute that can specify to which extant gene it corresponds. We now describe each event tag in details.

****<leaf>** tag:**

**Fig. 1.**
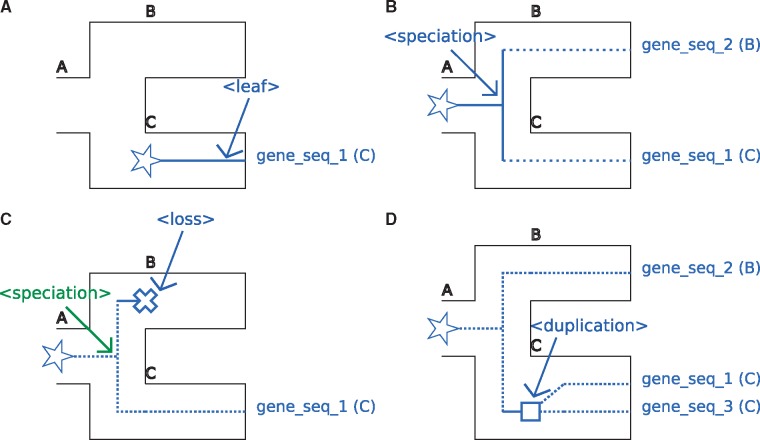
A. Representation of the <leaf> tag. B. Representation of the <speciation> tag. C. Representation of the <loss> tag. D. Representation of the <duplication> tag. The species tree is figured using black tube-like branches. The part of the gene tree the event occurs in is represented using plain lines. Additional parts of the gene tree are represented as dotted lines. Stars, squares and crosses respectively represent the beginning of a gene lineage, a gene duplication and a gene loss

The <leaf> tag indicates that the branch ends on a gene tree leaf; see Figure 1A. Note that the <leaf> tag also has a facultative geneName attribute that can specify to which extant gene it corresponds.

Associated recGeneTreeXML code:

<clade>

<name>gene_seq_1</name>

<eventsRec>

<leaf speciesLocation=“C”></leaf>

</eventsRec>

</clade>

**<speciation> tag:**

The <speciation> tag describes a gene lineage undergoing a bifurcation due to a speciation; see Figure 1B.

Associated recGeneTreeXML code:

<clade>

<name>n1</name>

<eventsRec>

<speciation speciesLocation=“A”></speciation>

</eventsRec>

</clade>

****<loss>** tag:**

The <loss> tag describes the loss of a gene copy and is a terminal tag (as with the <leaf> tag, there can be no tag following this one). Typically, it can follow a speciation event. See Figure 1C for an example.

Associated recGeneTreeXML code:

<!–Example with end tag <leaf>–>

<clade>

<name>n1</name>

<eventsRec>

<speciation>speciesLocation=“A”> </speciation>

</eventsRec>

<clade>

<name>gene_seq_1</name>

<eventsRec>

<leaf speciesLocation=“C”></leaf>

</eventsRec>

</clade>

<clade>

<name>LOST</name>

<eventsRec>

<loss speciesLocation=“B”></loss>

</eventsRec>

</clade>

</clade>

****<duplication>** tag:**

The <duplication> tag represents a gene duplication inside a species tree branch; see Figure 1D.

Associated recGeneTreeXML code:

<clade>

<name>n1</name>

<eventsRec>

<duplication speciesLocation=“C”>

</duplication>

</eventsRec>

</clade>

****<branchingOut>** tag:**

**Fig. 2.**
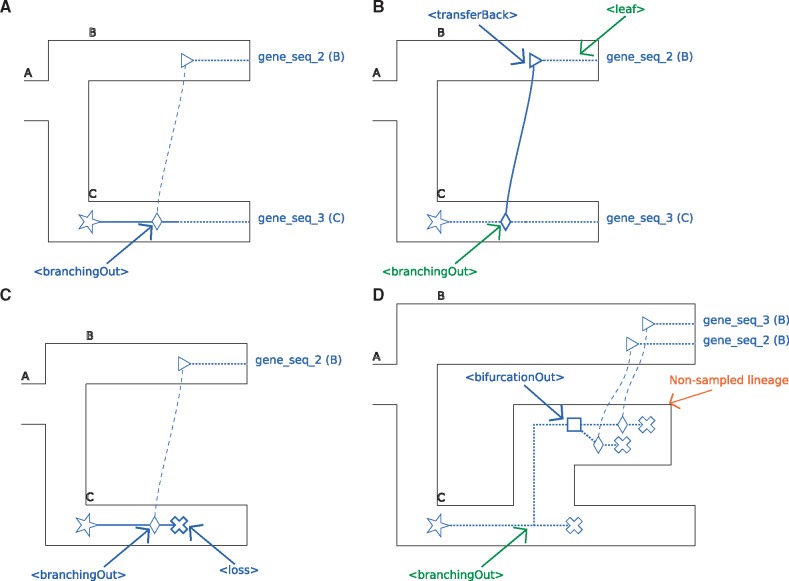
**A**. Representation of the <branchingOut> tag. **B**. Representation of the <transferBack> tag. **C**. Representation of a <branchingOut> tag followed by a <loss> tag. **D**. Representation of the <bifurcationOut> tag. These figure uses the same conventions as Figure 1 with the following additions. For the <bifurcationOut> tag (D.), which is specific of the model of Szöllősi *et al.* (2013b), extinct/unsampled lineages are represented as branches of the species tree that do not extend all the way to the right. Diamonds and triangles respectively represent a transfer leaving and entering a branch of the species tree (note that the transfers leave a branch of the species tree that corresponds to an extinct/unsampled lineage)

The <branchingOut> tag represents an event where a gene lineage splits and one gene copy exits the species tree branch while the other gene copy remains in the species branch. It actually is the first step of an horizontal gene transfer event: a gene lineage leaving a species tree branch; see Figure 2A. Figure 2C also represents the case of a <branchingOut> where the child that remained in the same species was lost (<loss> tag).

Associated recGeneTreeXML code:

<clade>

<name>n1</name>

<eventsRec>

<branchingOut speciesLocation=“C”>

</branchingOut>

</eventsRec>

</clade>

****<transferBack>** tag:**

The <transferBack> tag represents an horizontal gene transfer toward a branch of the species tree; see Figure 2B.

Associated recGeneTreeXML code:

<!–Example with end tag <leaf> –>

<clade>

<name>gene_seq_2</name>

<eventsRec>

<transferBack destinationSpecies=“B”>

</transferBack>

<leaf speciesLocation=“B”></leaf>

</eventsRec>

</clade>

****<bifurcationOut>** tag:**

The <bifurcationOut> tag represents a bifurcation in the species tree that would happen while the gene evolves along an unsampled/extinct species (i.e. one that is not represented in the species tree, see the <branchingOut> and <transferBack> tags above); see Figure 2D.

Associated recGeneTreeXML code:

<clade>

<name>n1</name>

<eventsRec>

<bifurcationOut></bifurcationOut>

</eventsRec>

</clade>

### 2.4 Note on the lateral gene transfer representation

**Fig. 3.**
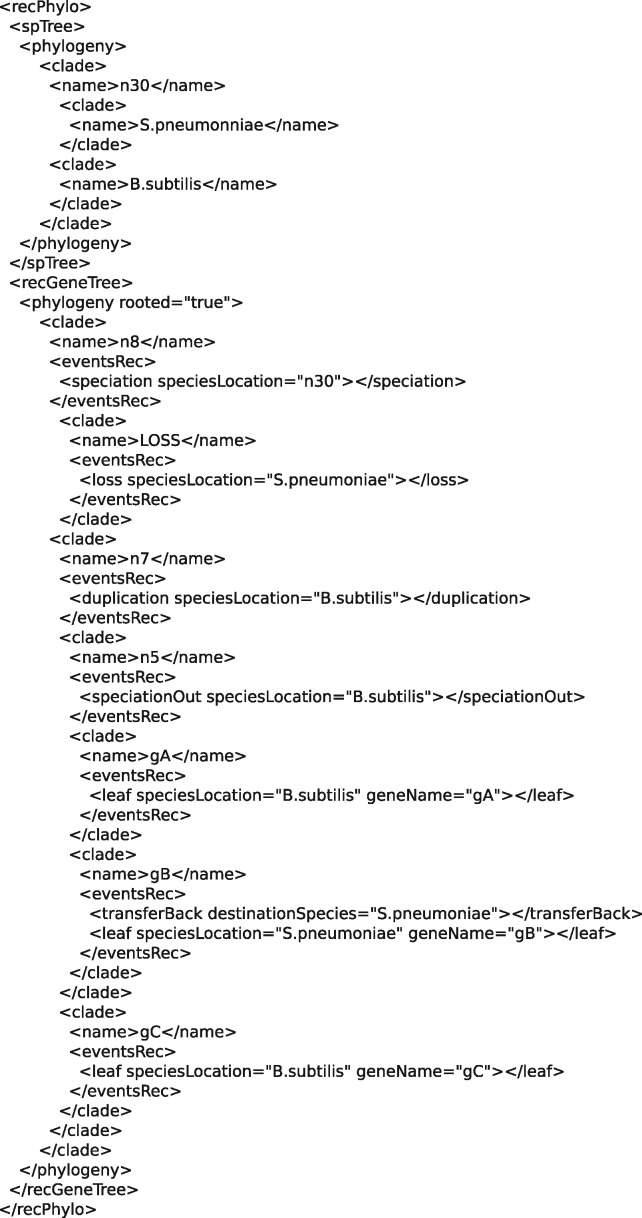
A <recPhylo> object containing a species tree and one reconciled gene tree

**Fig. 4.**
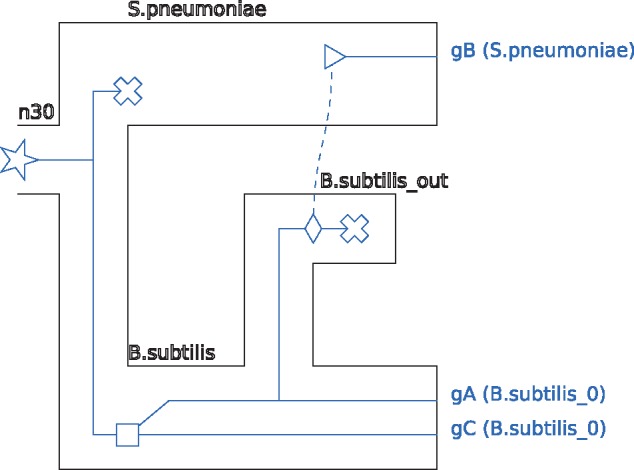
A visualization of the reconciled gene tree of Figure 3

A lateral gene transfer is represented in two steps: one that specifies the species where the transfer originates, and the other that specifies the species receiving the transfer. These two successive steps are respectively represented by the <branchingOut> and <transferBack> tags. See the different parts of Figure 2, along with Figures 3 and 4 for illustrations of these concepts.

### 2.5 recGeoXML

Geographical annotations can be indicated for gene and species tree nodes thanks to the <geography> tag. Such an annotation mainly consists in an area, KML information for displaying areas in GIS software and geographic information as defined in the usual PhyloXML grammar. An area (<area>) is specified by a name, a description, a value such as a support and a source (e.g. ‘observed’ or ‘inferred by Beast’).

### 2.6 recPhyloXML

recPhyloXML facilitates the packaging of several gene families reconciled to the same species tree. Its structure is fairly simple. A <recPhylo> root tag contains the following sequence:

- 0 to 1 species tree in recSpeciesTreeXML format, contained in the <spTree> tag.
- 1 to *n* gene family trees in recGeneTreeXML format, each defined in a separate <recGeneTree> tag.

<!– skeleton of a recphylo object with a species tree and two reconciled gene trees –>

<recPhylo>

<spTree>

<!– recSpeciesTreeXML species tree –>

…

</spTree>

<recGeneTree>

<!– first reconciled gene tree –>

…

</recGeneTree>

<recGeneTree>

<!– second reconciled gene tree –>

…

</recGeneTree>

</recPhylo>

A complete example of a <recPhylo> object containing a species tree and a reconciled gene tree can be seen in Figure 3 and a visualization of this reconciled gene tree can be seen in Figure 4.

## 3 Availability

A detailed description of the recPhyloXML format, as well as a schema definition file (This is a file formally describing the format, used by many XML tools.), is available at http://phylariane.univ-lyon1.fr/recphyloxml/. This website also presents a tool that can generate a visual representation of any reconciled tree or group of reconciled trees in the recPhyloXML format. The generated file is a.*svg* file, which easily allows for further manipulation, like changing the color scheme. This tool has been used to generate the basis for the figures showing reconciled gene trees in this manuscript.

The recPhyloXML format has already been implemented as an output option in the reconciliation software ALE (Szöllősi *et al.*, 2013a), in the Treerecs software [https://gitlab.inria.fr/Phylophile/Treerecs, this program corrects gene trees with a species tree using principles described in Noutahi *et al.* (2016) and Lafond *et al.* (2012)], in the reconciliation web server http://phylotoo2.appspot.com**/** (Zheng and Zhang, 2017), in the genome simulation software Zombi (https://github.com/AADavin/ZOMBI**)**, both as input and output options in the adjacency history computing software DeCoSTAR (Duchemin *et al.*, 2017).

Furthermore, scripts have been developed to convert the reconciliations produced by ecceTERA (Jacox *et al.*, 2016), NOTUNG (Durand *et al.*, 2006) and PrIME (Åkerborg *et al.*, 2009) into recPhyloXML, and a script for converting reconciliations produced by RANGER-DTL (Bansal *et al.*, 2018) is currently under development. Additional scripts are also available to convert a recPhyloXML reconciled tree in the Newick format, count the different events represented in a recPhyloXML file, combine different files into one or extract specific trees from a file. APIs have been written to import and export in recPhyloXML for the C++ library Bio++ (Gueguen *et al.*, 2013), for the python libraries ETE3 (Huerta-Cepas *et al.*, 2016) and Biopython (Cock *et al.*, 2009). All these scripts and APIs are available at https://github.com/WandrilleD/recPhyloXML.

## 4 Conclusion

With the growing number of available reconciliation models and pieces of software, it becomes crucial to be able to exchange and compare their results. recPhyloXML is a format that can accommodate many reconciliation features (dated/undated; with or without lateral gene transfers). It relies on an XML structure which is a standard format for nested data that already has multiple API libraries in various programming languages. We provide a detailed description of the recPhyloXML format on a website, along with a tool to visualize it.

We designed the format to be flexible in order to be able to create extensions that allow the representation of different forms of reconciliations. We are planning for future extensions for the format that would include a representation of the coalescent process that underlies ILS. recPhyloXML could also be extended to support gene conversion by a paralog or horizontal gene transfer with replacement.
